# Two Years, Many Clues: Diagnostic Journey and Treatment of Chronic Neurobrucellosis in an Elderly Woman

**DOI:** 10.7759/cureus.99774

**Published:** 2025-12-21

**Authors:** Pedro Vasques, Daniel Araújo, Andreia Paulos, Maria Lima, Ana Catarina Gonçalves, Evelise Ramos, João Pedro Caria, Andre Militão, Maria Isabel Casella

**Affiliations:** 1 Infectious Diseases, Unidade Local Saúde Arrábida, Setúbal, PRT; 2 Neurology, Unidade Local Saúde Arrábida, Setúbal, PRT; 3 Infectious Diseases, Centro Hospitalar de Lisboa Central, Lisbon, PRT

**Keywords:** central nervous system infections (cns), diagnosis, infectious diseases, neurobrucellosis, treatment

## Abstract

Neurobrucellosis is a rare complication of brucellosis, a zoonotic infection caused by *Brucella* spp. Its varied neurological manifestations make diagnosis difficult, often resulting in delays and increased morbidity. We present the case of a 61-year-old woman with recurrent neurological symptoms over two years, including confusion, gait imbalance, and cognitive decline. Despite extensive investigations showing ischemic changes on brain imaging, intrathecal IgG synthesis, and persistent cerebrospinal fluid (CSF) lymphocytic pleocytosis with initially sterile cultures, a presumptive diagnosis of primary central nervous system (CNS) vasculitis was made. After several hospital admissions and empirical treatments, *Brucella melitensis* was eventually isolated from the CSF, confirming chronic neurobrucellosis. Targeted antibiotic therapy led to significant clinical improvement. This case underscores the need to consider neurobrucellosis in patients with chronic or unexplained neurological symptoms, even when exposure history is unclear. Long incubation periods and nonspecific symptoms can make exposure difficult to recall and may cause clinicians to focus initially on more common etiologies.

## Introduction

Brucellosis has a worldwide distribution, with up to 500,000 cases diagnosed annually [1,2], and is endemic in the Mediterranean Basin, the Middle East, Central and South America, parts of sub-Saharan Africa, and South and Central Asia [3]. The bacteria primarily infect livestock and wildlife, while humans are incidental hosts who typically acquire the infection through contact with animal tissues or secretions, consumption of unpasteurized animal products, or inhalation of aerosols [3]. Of the dozen known *Brucella* species, four account for most human infections [3]: *Brucella melitensis* (from sheep/goats and the most virulent in humans), *Brucella abortus* (cattle), *Brucella suis* (swine), and *Brucella canis* (dogs). After entering the host, these organisms predominantly affect the reticuloendothelial system and use multiple mechanisms to evade immune detection.

Neurobrucellosis, estimated to occur in 4%-5% of brucellosis cases [1,2], is usually acquired through hematogenous spread [4]. Its underlying immunopathology involves meningeal granulomatosis and vasculitis, giving rise to a broad range of possible clinical presentations, including meningoencephalitis, transverse myelitis, peripheral neuropathy, and space-occupying lesion syndromes [2], often with a protracted course. Laboratory diagnosis relies on prolonged culture of this fastidious organism in blood, CSF, or tissue biopsy samples, along with serological evidence of infection [4,5].

There is no consensus on the optimal treatment for neurobrucellosis, but successful regimens typically involve prolonged combination antibiotic therapy with agents that have good central nervous system (CNS) penetration, such as ceftriaxone, rifampicin, cotrimoxazole, and doxycycline [6,7]. Sequelae are common due to delays in diagnosis and treatment. These vary depending on the clinical presentation and the CNS structures involved, but most often include motor deficits and sensorineural hearing or visual impairment [4,8].

## Case presentation

We present the case of a 61-year-old woman with a medical history of breast cancer treated with surgery, chemotherapy, and radiotherapy in 2006, with discharge from oncology follow-up in 2014; poorly controlled hypertension; chronic primary headaches for several decades treated with triptans and NSAIDs; and anxiety managed with benzodiazepines.

To better understand the case, a chronological summary of the most relevant emergency room (ER) visits and hospital admissions is presented below.

2021-2022

The patient, who was fully autonomous and living alone in Setúbal, had no known history of contact with livestock or consumption of unpasteurized dairy products. She first presented to the ER in June 2021 with a two-month history of headache, tinnitus, and malaise, along with episodes of fainting, gait imbalance, periods of confusion and incoherent speech, and, according to relatives, an unintentional 9 kg weight loss (approximately 10% of her body weight). Physical examination on admission was largely unremarkable except for mild confusion. There were no focal neurological deficits, including no changes in speech, coordination, or gait. Vital signs were mostly normal, except for mild tachycardia (105 bpm); the patient was normotensive, afebrile, and had a peripheral oxygen saturation of 98% on room air. Laboratory findings are summarized in Table 1.

**Table 1:** Blood tests and urinalysis on admission (June 2021) showing elevated inflammatory markers and leukocyturia, consistent with a urinary tract infection (UTI) HPF: high-power field.

Laboratory finding	Value	Reference range
Hemoglobin	11 g/dL	11.5-15 g/dL
Leukocyte count	13.400/μL	4.500-11.400/μL
C-Reactive protein	23.8 mg/dL	<0.5 mg/dL
Erythrocyte sedimentation rate	72 mm/h	1-20 mm/h
Urinalysis	250 leukocytes/HPF	<25 /HPF

Besides the laboratory results, a brain computed tomography (CT) scan showed a previous left cerebellar infarction, with no acute findings. She was admitted to the Infectious Diseases (ID) Department, where empirical ceftriaxone was started for a presumed urinary tract infection (UTI). This led to improvement in her neurocognitive symptoms and normalization of inflammatory markers after completing treatment. A whole-body CT scan excluded malignancy but revealed multiple enlarged right cervical lymph nodes considered pathologic based on number, size, and enhancement. Neurology recommended an electroencephalogram (EEG) and brain magnetic resonance imaging (MRI). The EEG showed no abnormalities, and the MRI, which was performed after hospital discharge, demonstrated ischemic changes in both cerebellar hemispheres (previously seen only on the left in the CT scan) and in the periventricular white matter.

Over the following two months, laboratory investigations showed persistent anemia (Hb 11 g/dL) and slightly elevated erythrocyte sedimentation rate (ESR; 47 mm/h) and ferritin (846 ng/mL), with negative infectious serologies (human immunodeficiency virus (HIV), hepatitis C virus (HCV), hepatitis B virus (HBV), Venereal Disease Research Laboratory (VDRL)) and negative autoimmunity markers (anti-dsDNA and ENA antibodies) (Table 2).

**Table 2:** Relevant blood test results from June to December 2021, showing persistently elevated inflammatory markers with negative infectious serologies and autoimmunity studies ds-DNA: double-stranded DNA, ENA: extractable nuclear antigen, HIV: human immunodeficiency virus, Ag p24: antigen p24, Ag HBs: hepatitis B virus surface antigen, HCV: hepatitis C virus.

Parameter	Value	Reference range
Hemoglobin	11 g/dL	11.5-15 g/dL
Erythrocyte sedimentation rate	47 mm/h	1-20 mm/h
Ferritin	846 ng/mL	30-200 ng/mL
Anti-ds-DNA	<0.5 UI/mL	<10 UI/mL
ENA antibodies	Negative	-
Anti-HIV-1/2 antibodies + Ag p24	Negative	-
Ag HBs	Negative	-
Anti-HCV antibodies	Negative	-
Total antibodies (IgM + IgG) for syphilis	Negative	-

A cervical CT scan was performed to further assess the noted lymphadenopathy and revealed Waldeyer’s ring hypertrophy. Biopsy of this area ruled out malignancy. Given her weight loss and persistent anemia, the patient underwent upper endoscopy, which showed mild gastritis with *Helicobacter pylori* infection, and a total colonoscopy, which was unremarkable. As part of the stroke workup, transthoracic echocardiography (TTE) and Holter monitoring were also conducted, with no significant abnormalities detected.

The patient was referred for Neurology outpatient follow-up and, over the next several months, underwent additional diagnostic testing. Transcranial Doppler and neck vessel ultrasound scans were unremarkable. A transesophageal echocardiogram (TEE) revealed a patent foramen ovale (PFO) with bidirectional shunting. A positron emission tomography (PET) scan showed increased metabolic activity in the nasopharynx, although malignancy had already been excluded on biopsy. A repeat brain and spinal cord MRI, performed eight months after the initial study, showed no new abnormalities. She was then referred for PFO closure, pending exclusion of chronic pulmonary embolism (PE) through a thoracic angio-CT scan.

During this period, the patient continued to experience headaches, dizziness, and gait imbalance, leading to four additional ER visits. In her final visit, in October 2022, she was found at home lying on the floor, with incoherent speech and urinary incontinence. On arrival to the ER, she had a temperature of 38 °C and reported memory difficulties and bilateral hand paraesthesias. A lumbar puncture (LP) was performed, and the cerebrospinal fluid (CSF) findings are shown in Table 3.

**Table 3:** CSF findings of LP at admission (October 2022): lymphocytic pleocytosis, apparently compatible with aseptic meningitis CSF: cerebrospinal fluid, PCR: polymerase chain reaction.

Parameter	Value	Reference range
CSF cell count	55 leukocytes/μL (80% lymphocytes)	<5 leukocytes/μL
Proteinorrhachia	206 mg/dL	15-40 mg/dL
Glycorrhachia	56 mg/dL	40-70 mg/dL
Glycemia	111 mg/dL	80-115 mg/dL
CSF multiplex PCR	Negative	-

During her stay in the ER, suprapubic tenderness was noted on physical examination, and her urine appeared cloudy. This prompted initiation of empirical ceftriaxone for a presumed UTI, along with concurrent aseptic meningitis.

She was admitted to the ID Department for continued evaluation. Urine culture later grew *Enterococcus faecium*, and antimicrobial therapy was adjusted to a seven-day course of linezolid after she developed an erythematous infusion reaction compatible with Red Man Syndrome during vancomycin administration. During this admission, and to further broaden the diagnostic workup, a repeat lumbar puncture was performed (Table 4), along with additional blood tests, including *Borrelia* spp. serology and peripheral blood immunoglobulin levels with immunophenotyping (Table 5).

**Table 4:** CSF findings of repeat LP (October 2022): persistent lymphocytic pleocytosis with no infectious agent identified and unremarkable anatomopathologic exam CSF: cerebrospinal fluid, ADA: adenosine deaminase, PCR: polymerase chain reaction.

Parameter	Value	Reference range
CSF cell count	70 leukocytes/μL (65% lymphocytes)	<5 leukocytes/μL
Proteinorrhachia	157 mg/dL	15-40 mg/dL
ADA	13 U/L	<9 U/L
Glycorrhachia	49 mg/dL	40-70 mg/dL
Glycemia	120 mg/dL	80-115 mg/dL
CSF multiplex PCR	Negative	-
*Mycobacterium tuberculosis* PCR	Negative	-
*Tropheryma whipplei *PCR	Negative	-
Bacterial culture	Sterile	-
Mycobacterial culture	Sterile	-
Anatomopathological exam	Unspecific lymphoplasmacytic infiltrate	-

**Table 5:** Peripheral blood immunoglobulin levels, immunophenotyping, and Borrelia serology (October 2022): no evidence of lymphoproliferative disease and further exclusion of another infectious etiology

Parameter	Value	Reference range
Immunoglobulin G	1140 mg/dL	700-1600 mg/dL
Immunoglobulin A	353 mg/dL	70-400 mg/dL
Immunoglobulin M	59.7 mg/dL	40-230 mg/dL
Kappa light chains	294 mg/dL	170-370 mg/dL
Lambda light chains	177 mg/dL	90-210 mg/dL
Peripheral blood immunophenotyping	Unremarkable	-
*Borrelia burgdorferi* antibodies - IgM	Negative	-
*Borrelia burgdorferi* antibodies - IgG	Negative	-

Repeat EEG remained normal. Angio-CT of the thorax was performed and could not definitively rule out PE, which was subsequently achieved by ventilation-perfusion scintigraphy.

2023

In January 2023, she continued to exhibit mild cognitive impairment, mainly characterized by executive dysfunction. An EEG performed in the outpatient setting revealed mild epileptiform activity for the first time, and the patient was started on levetiracetam.

In April, she returned to the ER with fever, headache, confusion, nausea, and vomiting; hence, another LP was performed (Table 6).

**Table 6:** LP at ER admission (April 2023): persistent lymphocytic pleocytosis, with no infectious agent identified (including Cryptococcus spp.) CSF: cerebrospinal fluid, LP: lumbar puncture, ER: emergency room, PCR: polymerase chain reaction.

Parameter	Value	Reference range
CSF cell count	60 leukocytes/μL (75% lymphocytes)	<5 leukocytes/μL
Proteinorrhachia	183 mg/dL	15-40 mg/dL
Glycorrhachia	45 mg/dL	40-70 mg/dL
Glycemia	132 mg/dL	80-115 mg/dL
CSF multiplex PCR	Negative	-
*Cryptococcus* antigen	Negative	-
Bacterial culture	Sterile	-

She was readmitted for further evaluation, again with a working diagnosis of aseptic meningitis. During this stay, she continued to exhibit mild confusion and intermittent, self-limited fever without an identifiable infectious source. Blood and urine cultures remained negative. Following the Neurology consultation, additional investigations were performed, including another EEG, repeat brain MRI, and a new LP with expanded testing as recommended (Table 7). These studies showed findings similar to previous evaluations, notably persistent lymphocytic pleocytosis and, importantly, evidence of intrathecal IgG synthesis on oligoclonal band testing. This latter result represented a key milestone in the diagnostic process.

**Table 7:** Repeat LP (April 2023): persistent lymphocytic pleocytosis with unremarkable microbiological and anatomopathologic results; autoimmune CNS disease further excluded, but now showing intrathecal IgG synthesis, a key finding contributing to the diagnosis CSF: cerebrospinal fluid, LP: lumbar puncture, CNS: central nervous system. Note: CSF flow cytometry, oligoclonal bands, and antineuronal antibody panel were performed outside the hospital.

Parameter	Value	Reference range
CSF cell count	50 leukocytes/μL (75% lymphocytes)	<5 leukocytes/μL
Proteinorrhachia	209 mg/dL	15-40 mg/dL
Glycorrhachia	45 mg/dL	40-70 mg/dL
Glycemia	132 mg/dL	80-115 mg/dL
Bacterial culture	Sterile	-
Mycobacterial culture	Sterile	-
Anatomopathologic exam	No signs of malignancy	-
Flow cytometry	CD4 predominance, no monoclonality	-
Oligoclonal bands	Intrathecal IgG synthesis	-
Antineuronal antibody panel	Negative	-

She was discharged after showing clinical improvement but was readmitted to the ID Department shortly thereafter with fever, vomiting, confusion, dysarthria, and quadriparesis (MRC 3/5-4/5). The LP findings during this admission showed slight differences compared to prior evaluations (Table 8), and an extensive panel of additional tests was performed to further broaden the diagnostic workup (Table 9).

**Table 8:** LP at readmission (May 2023): no pleocytosis observed; only hyperproteinorrachia present CSF: cerebrospinal fluid, LP: lumbar puncture.

Parameter	Value	Reference range
CSF cell count	<5 leukocytes/μL (100% lymphocytes)	<5 leukocytes/μL
Proteinorrhachia	162 mg/dL	15-40 mg/dL
Glycorrhachia	62 mg/dL	40-70 mg/dL
Glycemia	147 mg/dL	80-115 mg/dL
Bacterial culture	Sterile	-
Mycobacterial culture	Sterile	-
Mycological culture	Sterile	-

**Table 9:** Additional tests requested at readmission (infectious serologies and autoimmunity studies; May 2023): all tested etiologies apparently excluded, including a negative Huddleson reaction CMV: cytomegalovirus, EBV-CA: Epstein-Barr virus capsid antigen, EBNA: Epstein-Barr nuclear antigen, ANA: antinuclear antibodies, ENA: extractable nuclear antigen, ds-DNA: double-stranded DNA, PR3: proteinase-3, MPO: myeloperoxidase.

Parameter	Value	Reference range
Huddleson reaction	Negative	-
CMV - IgM	Negative	<0.7 UI/mL (negative); >0.9 UI/mL (positive)
CMV - IgG	82 UI/mL	<4 UI/mL (negative); >6 UI/mL (positive)
EBV - CA IgM	<10 UI/mL	<20 UI/mL (negative); >40 UI/mL (positive)
EBV - CA IgG	302 UI/mL	<20 UI/mL (negative); >20 UI/mL (positive)
EBNA - IgG	57 UI/mL	<5 UI/mL (negative); >20 UI/mL (positive)
*Mycoplasma pneumoniae* - IgM	<0.1 Ua/mL	<10 Ua/mL (negative); >10 Ua/mL (positive)
*Mycoplasma pneumoniae* - IgG	<0.1 Ua/mL	<10 Ua/mL (negative); >10 Ua/mL (positive)
*Toxoplasma* - IgM	Negative	<0.55 UI/mL (negative); >0.65 UI/mL (positive)
*Toxoplasma* - IgG	Negative	<4 UI/mL (negative); >8 UI/mL (positive)
CSF - arboviruses IgM	Negative	-
CSF - arboviruses IgG	Negative	-
ANA	Negative	-
ENA	Negative	-
Anti-ds-DNA	<0.5 UI/mL	<10 UI/mL (negative); >15 UI/mL (positive)
Anti-cardiolipin - IgM	3.4 UI/mL	<10 UI/mL (negative); >40 UI/mL (positive)
Anti-cardiolipin - IgG	<0.5 UI/mL	<10 UI/mL (negative); >40 UI/mL (positive)
Lupus anticoagulant	Not observed	-
Anti-glycoprotein I antibodies - IgM	<2.9 UI/mL	<7 UI/mL (negative); >10 UI/mL (positive)
Anti-glycoprotein I antibodies - IgG	<0.6 UI/mL	<7 UI/mL (negative); >10 UI/mL (positive)
Anti-PR3	<0.2 UI/mL	<2 UI/mL (negative); >3 UI/mL (positive)
Anti-MPO	<0.2 UI/mL	<2 UI/mL (negative); >5 UI/mL (positive)

Later in her stay, *Escherichia coli* was isolated from a urine culture obtained during a recurrence of fever, and she completed a one-week course of cefuroxime based on susceptibility testing. Although the fever resolved, her fluctuating neurological deficits persisted. Given the previously documented intrathecal IgG synthesis, the case was again discussed with Neurology. At this stage, primary CNS vasculitis became the leading diagnostic hypothesis, and the patient was transferred to the Neurology ward in June.

**Figure 1:**
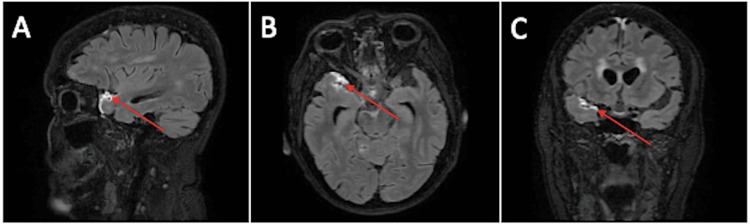
(A) Sagittal, (B) axial, and (C) coronal gadolinium-enhanced FLAIR images (June 2023) Enhancement of the anterior aspect of the right Sylvian fissure is visible (red arrows), supplied by the homolateral MCA inferior division. FLAIR: fluid-attenuated inversion recovery, MCA: middle cerebral artery.

**Figure 2:**
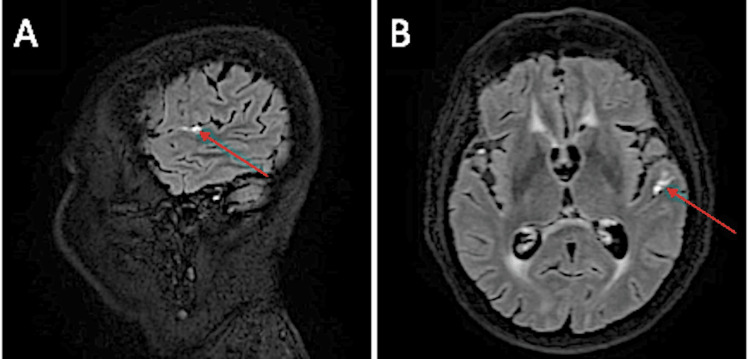
(A) Sagittal and (B) axial gadolinium-enhanced FLAIR images (June 2023) Enhancement of the posterior aspect of the left Sylvian fissure (red arrows), supplied by the superior temporal division of the left MCA inferior branch. FLAIR: fluid-attenuated inversion recovery, MCA: middle cerebral artery.

Brain angio-MRI demonstrated leptomeningitis and mural enhancement involving the inferior division of the right middle cerebral artery (MCA) (Figure 1) and the superior temporal branch of the inferior division of the left MCA (Figure 2). Digital subtraction angiography, performed at another hospital, showed a “string-of-beads” appearance in the M3 segment of the right MCA, findings supportive of vasculitis. A repeat LP revealed persistent hyperproteinorrachia and lymphocytic pleocytosis, and CSF flow cytometry remained unremarkable (Table 10).

**Table 10:** LP performed at the Neurology Department (June 2023): lymphocytic pleocytosis observed once again CSF: cerebrospinal fluid, LP: lumbar puncture. Note: CSF flow cytometry was performed outside the hospital.

Parameter	Value	Reference range
CSF cell count	115 leukocytes/μL (70% lymphocytes)	<5 leukocytes/μL
Proteinorrhachia	313 mg/dL	15-40 mg/dL
Glycorrhachia	64 mg/dL	40-70 mg/dL
Glycemia	100 mg/dL	80-115 mg/dL
Bacterial culture	Sterile	-
Flow cytometry	CD4 lymphocyte predominance, no monoclonality	-

It is also worth noting that she developed an episode of acute urinary retention in the context of yet another *E. coli* UTI, accompanied by mild bilateral hydronephrosis on ultrasound. Directed cotrimoxazole therapy and urinary catheterization, with successful removal after five days, promptly resolved this complication.

Suspected primary CNS vasculitis prompted high-dose corticosteroid therapy (methylprednisolone pulses followed by an oral prednisolone taper). However, her condition deteriorated, with worsening confusion and psychomotor slowing. During this period, EEG documented a non-convulsive status epilepticus, which resolved after lacosamide was added to her ongoing levetiracetam regimen. Cyclophosphamide was then initiated, and she completed three monthly cycles between August and October.

**Figure 3:**
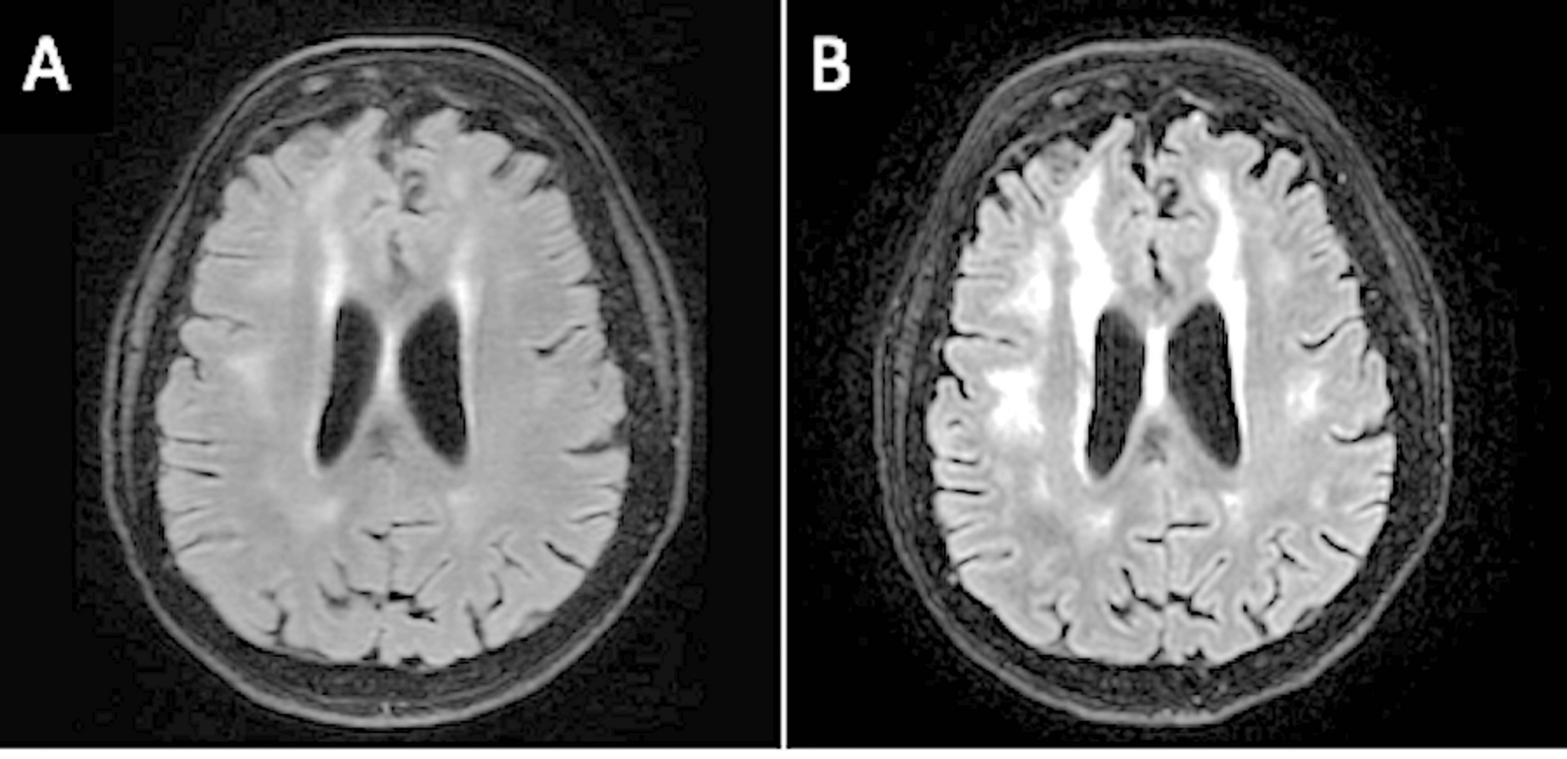
Axial FLAIR images from (A) June 2023 and (B) September 2023 Poorly defined areas of subcortical and periventricular hyperintensities mainly located in the frontoparietal white matter are visible, with significant worsening between the two exams. FLAIR: fluid-attenuated inversion recovery.

Neurological deficits persisted during this time, including fluctuating quadriparesis, gait ataxia, and intermittent episodes of encephalopathy. MRI performed at the end of the first cyclophosphamide cycle (August) showed worsening T2 hyperintensities and juxtacortical frontoparietal white-matter lesions, consistent with microvascular ischemic changes. She also received a second methylprednisolone pulse during the second cyclophosphamide cycle in September. A repeat MRI following this second cycle demonstrated findings similar to the previous study (Figure 3). Two additional LPs, performed at the end of the second and third cycles, respectively, continued to show persistent lymphocytic pleocytosis and elevated CSF protein levels (Tables 11, 12).

**Table 11:** LP at the end of the second cyclophosphamide cycle (September 2023): persisting lymphocytic pleocytosis with immunosuppressive therapy initiated to treat presumed primary CNS vasculitis CSF: cerebrospinal fluid, LP: lumbar puncture, CNS: central nervous system.

Parameter	Value	Reference range
CSF cell count	85 leukocytes/μL (70% lymphocytes)	<5 leukocytes/μL
Proteinorrhachia	217 mg/dL	15-40 mg/dL
Glycorrhachia	57 mg/dL	40-70 mg/dL
Glycemia	81 mg/dL	80-115 mg/dL
Bacterial culture	Sterile	-

**Table 12:** LP at the end of the second cyclophosphamide cycle (September 2023): persistent lymphocytic pleocytosis despite initiation of immunosuppressive therapy for presumed primary CNS vasculitis CSF: cerebrospinal fluid, LP: lumbar puncture.

Parameter	Value	Reference range
CSF cell count	28 leukocytes/μL (100% lymphocytes)	<5 leukocytes/μL
Proteinorrhachia	80 mg/dL	15-40 mg/dL
Glycorrhachia	65 mg/dL	40-70 mg/dL
Glycemia	87 mg/dL	80-115 mg/dL
Bacterial culture	Brucella melitensis	-

In the final lumbar puncture, *B. melitensis* was isolated from the CSF culture and subsequently confirmed by serology (Table 13).

**Table 13:** Brucella serologic tests following positive CSF culture (October 2023): results confirming the unexpected diagnosis of neurobrucellosis CSF: cerebrospinal fluid. Note: Blood and CSF *Brucella* IgG, IgA, and immunocapture tests were performed outside the hospital.

Parameter	Value	Reference range
Rose Bengal test	Positive	-
Brucella - IgA (blood)	Positive	-
Brucella - IgG (blood)	Positive	
Brucella - IgA (CSF)	Positive	-
Brucella - IgG (CSF)	Positive	-
Immunocapture test	1:2560	-

The diagnosis of chronic neurobrucellosis was established, and targeted antibiotic therapy was initiated, consisting of intravenous (IV) ceftriaxone 2 g every 12 hours for six weeks, oral rifampicin 600 mg daily, and oral doxycycline 100 mg twice daily for at least 12 weeks.

In November, the patient developed right leg swelling and pain, and deep venous thrombosis (DVT) of the right lower limb was confirmed by angio-CT scan. Anticoagulation with enoxaparin was started, resulting in significant clinical improvement.

After six weeks of IV antibiotics, she showed marked improvement, regaining full orientation, sustained attention, and fluent, coherent speech. Postural control and motor coordination also improved, allowing her to stand without assistance, though only for brief periods. She was discharged to a convalescent care unit for motor rehabilitation to regain functional autonomy. Follow-up appointments were scheduled with both Neurology and ID, although the latter was subsequently missed. Outpatient medications included rifampicin 600 mg daily and doxycycline 100 mg twice daily for neurobrucellosis; prednisolone 2.5 mg daily and cotrimoxazole 800/160 mg three times per week for corticosteroid taper and chemoprophylaxis; rivaroxaban 20 mg daily for right lower limb DVT; amitriptyline 10 mg for chronic headache; levetiracetam 1500 mg twice daily; and risperidone 1 mg plus trazodone 150 mg at night to manage agitation, particularly in the context of corticosteroid therapy. She was also advised to monitor for dysuria, suprapubic pain, or decreased urine flow, given her history of recurrent UTIs and a prior episode of urinary retention.

2024

Upon reassessment in a Neurology appointment in February, the patient had no deficits apart from a grade 4/5 proximal paresis with flexion of the right thigh, mild right-predominant dysmetria on finger-to-nose test (sequelae of previous cerebellar strokes), and a slight gait imbalance, with ambulation only possible with unilateral support from a caregiver. By this time, the patient was on her 16th week of treatment with rifampicin and doxycycline, and repeat serologic tests and LP were planned to monitor treatment response.

Accordingly, blood tests were repeated in April (Table 14), followed by another LP in May (Table 15).

**Table 14:** Repeat serologic tests and erythrocyte sedimentation rate (ESR) (April 2024; 21 weeks of treatment): ESR normalized, suggesting therapeutic efficacy

Parameter	Value	Reference range
Huddleson reaction	Positive	-
Immunocapture test	1:5120	-
Erythrocyte sedimentation rate	20 mm/h	1-20 mm/h

**Table 15:** Repeat LP (May 2024; 28 weeks of treatment): persistent lymphocytosis despite ongoing therapy CSF: cerebrospinal fluid, PCR: polymerase chain reaction. Note: CSF *Brucella* IgG, IgA, and IgM, and *Brucella* DNA PCR were performed outside the hospital.

Parameter	Value	Reference range
CSF cell count	20 leukocytes/μL (no cell predominance)	<5 leukocytes/μL
Proteinorrhachia	67 mg/dL	15-40 mg/dL
Glycorrhachia	51 mg/dL	40-70 mg/dL
Glycemia	147 mg/dL	80-115 mg/dL
Bacterial culture	Sterile	-
Brucella IgM	Negative	-
Brucella IgA	Positive	
Brucella IgG	Positive	
Brucella DNA PCR	Positive	-

Due to persistent pleocytosis despite clinical improvement, presumed therapeutic failure was considered, and the patient was readmitted to the Neurology Department in August for a second six-week course of ceftriaxone. The case was reviewed again with ID, who recommended continuing rifampicin and doxycycline without a defined treatment duration. A LP performed during this admission showed significant improvement, as did the repeat serologic tests (Table 16).

**Table 16:** Repeat LP and blood serologic tests after new course of IV ceftriaxone (August 2024; 42 weeks of treatment): significant improvement as seen by reduction in cell count CSF: cerebrospinal fluid, LP: lumbar puncture. Note: CSF *Brucella *DNA PCR and immunocapture test were performed outside the hospital.

Parameter	Value	Reference range
CSF cell count	8 leukocytes/μL (no cell predominance)	<5 leukocytes/μL
Proteinorrhachia	77 mg/dL	15-40 mg/dL
Glycorrhachia	41 mg/dL	40-70 mg/dL
Glycemia	115 mg/dL	80-115 mg/dL
Bacterial culture	Sterile	-
Brucella DNA PCR (CSF)	Positive	-
Huddleson reaction	Positive	-
Immunocapture test	1:5120	-

2025

Clinically, the patient remained stationary, with some gait imbalance still requiring support. New LP in January finally showed no pleocytosis, although still with mildly elevated protein levels (Table 17).

**Table 17:** Repeat LP (January 2025; 64 weeks of treatment): no pleocytosis observed after prolonged therapy CSF: cerebrospinal fluid, PCR: polymerase chain reaction, LP: lumbar puncture. Note: CSF *Brucella *DNA PCR was performed outside the hospital.

Parameter	Value	Reference range
CSF cell count	<5 leukocytes/μL	<5 leukocytes/μL
Proteinorrhachia	59 mg/dL	15-40 mg/dL
Glycorrhachia	51 mg/dL	40-70 mg/dL
Glycemia	Unknown	80-115 mg/dL
Bacterial culture	Sterile	-
Brucella DNA PCR (CSF)	Negative	-

She is scheduled for clinical evaluations every three months, with a repeat lumbar puncture planned in one year if she remains clinically stable. Having been considered cured, rifampicin and doxycycline were discontinued in May 2025, after 80 weeks of treatment. No treatment-related side effects were reported by the patient during this period.

## Discussion

The patient’s long-standing, nonspecific neurological symptoms, sequelae of prior infarctions on CT and MRI, and history of weight loss prompted an extensive investigation. Unremarkable stroke workup, exclusion of cancer relapse, lymphoproliferative disorders, autoimmune disease, and multiple infectious etiologies initially left a diagnostic dead end, with repeated UTI diagnoses and later episodes of concurrent aseptic meningitis. In hindsight, the multiple courses of ceftriaxone for presumed UTI likely contributed to intermittent symptom improvement and relapse, resulting in numerous ER visits and hospital admissions and further delaying the diagnosis of neurobrucellosis. It is also likely that the Huddleson reaction performed a few months before *Brucella* isolation was a false negative, either due to a prozone effect or because the test detects only agglutinating antibodies. In chronic, long-standing forms of the disease, non-agglutinating antibodies tend to predominate [9-11]. Persistent lymphocytic pleocytosis across multiple LPs was the key finding that eventually guided the diagnosis, after repeated microbiological, anatomopathologic, serological, and molecular CSF exams showed nothing except intrathecal IgG synthesis. This finding initially suggested primary CNS vasculitis, a hypothesis further supported by DSA findings. After clinical and radiological worsening under immunosuppressive therapy, initially appearing paradoxical, *B. melitensis* was unexpectedly isolated from a CSF culture (after multiple prior LPs). Serological studies subsequently corroborated this result, which, despite initial skepticism, is definitive: CSF culture is a gold standard for neurobrucellosis diagnosis, albeit with a sensitivity of only 35%-45% and high variability across studies [8,12]. Positive Rose Bengal testing, along with immunocapture and ELISA for IgG and IgA in both blood and CSF, provided strong support for the diagnosis, which is essentially one of exclusion. CSF serology is highly specific for neurobrucellosis, and both ELISA and immunocapture assays are less prone to prozone effects, allowing detection of non-agglutinating antibodies [13-15].

Recommended treatment involves a prolonged combination of antibiotics, typically four to six weeks of IV ceftriaxone followed by doxycycline plus rifampicin for at least 12 weeks, sometimes extending to 24 weeks. Prolonged combination therapy is essential to prevent relapse, although supporting evidence remains limited [6,16].

Monitoring response to therapy generally involves repeated clinical assessment, serology, and CSF evaluation, with expected resolution of neurological symptoms and CSF lymphocytic pleocytosis [7,17]. Interestingly, a subsequent positive Huddleson reaction supported the hypothesis of a prior false-negative result due to a prozone effect, even though the test has no role in monitoring treatment response. No standardized serological criteria exist for defining eradication, treatment failure, relapse, or evolution to focal disease, as antibody levels vary widely across patients [18]. A twofold rise in immunocapture antibody titers is inconclusive, and the lack of standardization in many assays, including in-house tests, further complicates interpretation. In endemic areas or among patients with occupational exposure, higher antibody levels are expected in contacts, past infections, and treated individuals compared with patients from non-endemic areas or without occupational risk [19,20], underscoring the central role of clinical assessment and CSF analysis in monitoring treatment response.

## Conclusions

This case highlights the importance of considering neurobrucellosis in the differential diagnosis of unexplained neurological syndromes, even when it remains a diagnosis of exclusion. Early recognition and targeted therapy are essential to reduce mortality and prevent irreversible neurological damage. The potential for relapse during follow-up, combined with the typically prolonged and sometimes indefinite treatment duration, also underscores the inherent limitations of case reports involving this disease, as the clinical course may not be fully concluded. Because most of what is known about neurobrucellosis comes from case reports and small retrospective studies, the absence of standardized diagnostic criteria, established treatment regimens, and clear monitoring protocols becomes more understandable, a reflection of the elusive nature of this condition. We hope this case adds a meaningful contribution to the existing literature, given the extensive two-year investigation preceding *Brucella* isolation in a CSF culture sample, despite an earlier false-negative serology.
